# Biochar Enhances Soil Resource Availability and Suppresses Microbial Metabolism Genes in the Rhizosphere of Wheat

**DOI:** 10.3390/life13091843

**Published:** 2023-08-31

**Authors:** Xin Gong, Sixian Li, Zelu Wu, Yousef Alhaj Hamoud, Hiba Shaghaleh, Yusef Kianpoor Kalkhajeh, Chenxiao Si, Lin Zhu, Chao Ma

**Affiliations:** 1 Anhui Province Key Lab of Farmland Ecological Conservation and Pollution Prevention, Anhui Province Engineering and Technology Research Center of Intelligent Manufacture and Efficicent Utilization of Green Phosphorus Fertilizer, College of Resources and Environment, Anhui Agricultural University, Hefei 230036, China; xgong@njau.edu.cn (X.G.);; 2 Key Laboratory of Urban Environment and Health, Ningbo Observation and Research Station, Institute of Urban Environment, Chinese Academy of Sciences, Xiamen 361021, China; 3 College of Hydrology and Water Resources, Hohai University, Nanjing 210098, China; 4 College of Environment, Hohai University, Nanjing 210098, China; 5 College of Science and Technology, Wenzhou-Kean University, Wenzhou 325060, China

**Keywords:** biodiversity, community composition, functional diversity, metagenome, rhizosphere effects, straw biochar

## Abstract

Despite the well-documented role of biochar in promoting soil quality and crop productivity, the underlying biological mechanisms remain poorly understood. Here, we explored the effects of straw biochar on soil microbiome in the rhizosphere from wheat using metagenomic sequencing. Our results showed that straw return decreased the yields of wheat, while the straw biochar return increased the wheat yields. Further, both the richness and community composition confirmed different effects of the straw return and straw biochar return. The straw biochar return also resulted in greater rhizosphere effects from wheat, represented by resource availability, including soil organic carbon, soil total nitrogen, available phosphorus, and available potassium. The rhizosphere effects from wheat, represented by microbial metabolism genes involved in carbon, nitrogen, phosphorus, and potassium cycling, however, were decreased by straw biochar returning. In addition, the rhizosphere effects from nitrogen content and the nitrogen cycling genes showed negative relationships with wheat yields. Together, these results revealed that straw biochar enhanced soil resource availability but suppressed microbial metabolism genes in the rhizosphere from wheat, supporting the idea that straw biochar serves as a nutrient pool for crops.

## 1. Introduction

The rhizosphere is an active and dynamic interface essential for the well-being of plants [1,2]. Plants take up water and nutrients from the rhizosphere, whereas their roots secrete a variety of compounds into the interface, causing changes in the physiochemical and biological properties of the surrounding bulk soil [3]. The corresponding differences between the rhizosphere and bulk soil are known as “rhizosphere effects” that play crucial roles in determining soil biogeochemical processes [4]. Despite the growing recognition regarding the overall rhizosphere effects on soil biogeochemistry [5,6], little is known about the rhizosphere effects on soil microbial genomes that are the base for soil nutrient cycling [7]. 

Biochar is regularly used to improve soil quality and potentially mitigate global change [8,9]. Straw biochar can promote crop productivity by not only enhancing the uptake of soil available resources but also driving soil microbiome [10,11]. Despite the enhancements of the metabolic potential and subsequent soil available resources after biochar application, the underlying mechanisms are yet unknown [12,13]. Therefore, it is imperative to explore the shift of microbiomes in the rhizosphere, which helps to understand the rhizosphere effects on soil nutrients in biochar-amended soils. 

The effects of biochar on the diversity and community composition of soil and rhizosphere microbiota were documented [14]. The underlying effects of biochar on the microbial metagenome, however, were not clear. The microbial metagenome was the most relevant part of the microbial functional traits in determining nutrient cycling, which might be involved in the biochar effects. For example, a meta-analysis revealed that biochar could increase the uptake of phosphorus for plants but the phosphorus cycling genes were not elusive [11]. It is also noted that the metagame approach provides a holistic way to analyze the microbial community without PCR bias [15], which might be a benefit for understanding both microbial taxonomy and functions [16].

Here, we conducted a field experiment to investigate the rhizosphere effects of wheat under straw biochar applications (See Supplementary Information for more details). Briefly, three treatments were set up in triplicates, including no straw application (control, CK), straw cut into 5 cm lengths and returned to the field (straw return, SR), and straw transformed to biochar and returned to the field (straw biochar return, SBR). Bulk and rhizosphere soil sampling, soil analyses, and metagenome sequencing followed [12]. The rhizosphere effects were quantified as the magnitude of differences between the rhizosphere and bulk soils relative to the bulk soil [4]. We hypothesized that straw biochar application would stimulate the rhizosphere effects in both soil available resources and soil microbiome, thus promoting the yields of wheat.

## 2. Materials and Methods

### 2.1. Site Description

The field experiment was located in Mengcheng County, Anhui Province, China (32°13′ N, 116°37′ E). This region has a mean annual temperature and precipitation of about 16.5 °C and 822 mm, respectively. Lime concretion black soil is the dominant soil type in this area with pH 5.80; organic matter, 14.20 g kg^−1^; total N, 0.98 g kg^−1^; available P, 23.8 mg kg^−1^; and available K, 98.0 mg kg^−1^. A typical winter-wheat–summer-maize rotation is the main cropping system at this site. 

### 2.2. Experimental Design

This study employed a randomized complete block design with three replicates. The randomization of experimental plots was carried out via the RANDBETWEEN function in excel. Each plot had 6 m (length) × 5 m (width) (30 m^2^) dimensions. Three treatments were implemented at the research station including (1) no straw application (CK), (2) straw was cut into 5 cm and returned to the field (SR), and (3) straw was transformed into biochar and returned to the field (SBR). In straw amendment treatment, wheat straw and maize straw were applied in maize and wheat seasons at the rates of 6000 and 7500 kg ha^−1^, respectively. Biochar was prepared from wheat and maize straws by pyrolysis at 450 °C for 4 h in a N_2_ atmosphere (it was anticipated that 30% by mass of the crop straw would be converted to biochar). Biochar was applied in maize and wheat seasons at the rates of 2000 and 2500 kg ha^−1^, respectively. Details of the chemical NPK fertilization are summarized in Table 1. 

Soil samples were taken after the wheat heading stage. Loose soil was removed from the roots by kneading and shaking, as well as by patting the roots on the back of a gloved hand. The firmly adhered soil to the roots was referred to as rhizosphere soil. For individual plots, representative bulk soils composed of five subsamples were collected at a depth of 0~15 cm. All non-rhizosphere and rhizosphere soil samples were air-dried in darkness at room temperature and then crushed manually to determine soil properties. Further, fresh soil samples were stored at −80 °C for metagenomics analysis.

For each treatment, the panicles at three different locations with an area of 1 m^2^ were counted to measure the number of panicles per square meter. The panicles were then harvested to determine the yield.

### 2.3. Soil Physicochemical and Metagenomics Analyses

Soil organic carbon was determined by the K_2_Cr_2_O_7_-H_2_SO_4_ oxidation method [17]. Soil total nitrogen was determined by the Kjeldahl method following sample digestion [18]. Soil-available phosphorus was extracted by 0.5 M sodium bicarbonate and determined by the molybdenum blue method [19]. The soil content of potassium was determined by flame photometry (6400A, INESA, Shanghai, China) after extraction with 1 M ammonium acetate [20].

DNA was extracted from the soil samples in duplicate using the MoBio PowerSoil kit (MOBIO) according to the manufacturer’s protocol. DNA yields of the 10 samples were between 1.0 and 2.5 mg, as quantified using the Quant-iT Picogreen dsDNA HS assay kit (Invitrogen, Waltham, MA, USA). Sequencing was performed using HiSeq 3000/4000 SBS Kits (Illumina) at Majorbio, Inc., Shanghai, China. Raw reads (150 bp in length) were trimmed to remove low-quality reads that contained ambiguous nucleotides or had a Phred score lower than 30 [21]. In total, 1,227,013,238 clean reads were generated with an average of 68,167,402 reads per sample (Table S1). Raw sequences were deposited in NCBI with the accession number PRJNA898266.

The clean reads were assembled using MEGAHIT [22]. Contigs with lengths longer than 800 bps were selected for downstream analysis. Prodigal was used for gene prediction. Then, the non-redundant gene catalog was constructed using CD-HIT (identity 95%, coverage 90%) [23,24]. Bowtie2 was used to map the clean reads to each gene (95% identity) for the calculation of the relative abundance of each gene. Taxonomic annotation was performed using DIAMOND based on the NCBI NR database [25]. Functional annotation was performed using DIAMOND based on the databases of KEGG, eggNOG, and CAZy.

### 2.4. Data Processing and Analysis

We calculated the rhizosphere effect as the difference in each soil variable between rhizosphere soil and bulk soil according to Ding et al. [26]:

(1) $$\mathrm{Rhizosphere}\;\mathrm{effect}=(C_{\mathrm{rhizosphere}\_\mathrm{soil}}-C_{\mathrm{bulk}\_\mathrm{soil}})/C_{\mathrm{bulk}\_\mathrm{soil}}$$

where C_rhizosphere_soil_ represents the total soil gene, archaea, bacteria, viruses, soil resources, and soil biogeochemical cycling genes in the rhizosphere soils; and C_bulk_soil_ represents the corresponding index in the bulk soils.

All statistical analyses were performed using R 4.1.1 [27]. The difference in rhizosphere effects on the diversity of total soil genes, archaea, bacteria, viruses, soil resources, and soil biogeochemical cycling genes was tested using one-way ANOVA. The effects of different rates of straw application on the average rhizosphere were explored via an independent-sample t-test. The relationship between the microbial community dissimilarity and the straw application was evaluated using non-metric multidimensional scaling analyses (NMDS) in the vegan package. A permutation multivariate analysis of variance (PerMANOVA) using Bray-Curtis distances was applied to examine the differences in the contributions of compartments (bulk vs. rhizosphere) and straw application (CK, SR, and SBR). 

## 3. Results

**Figure 1:**
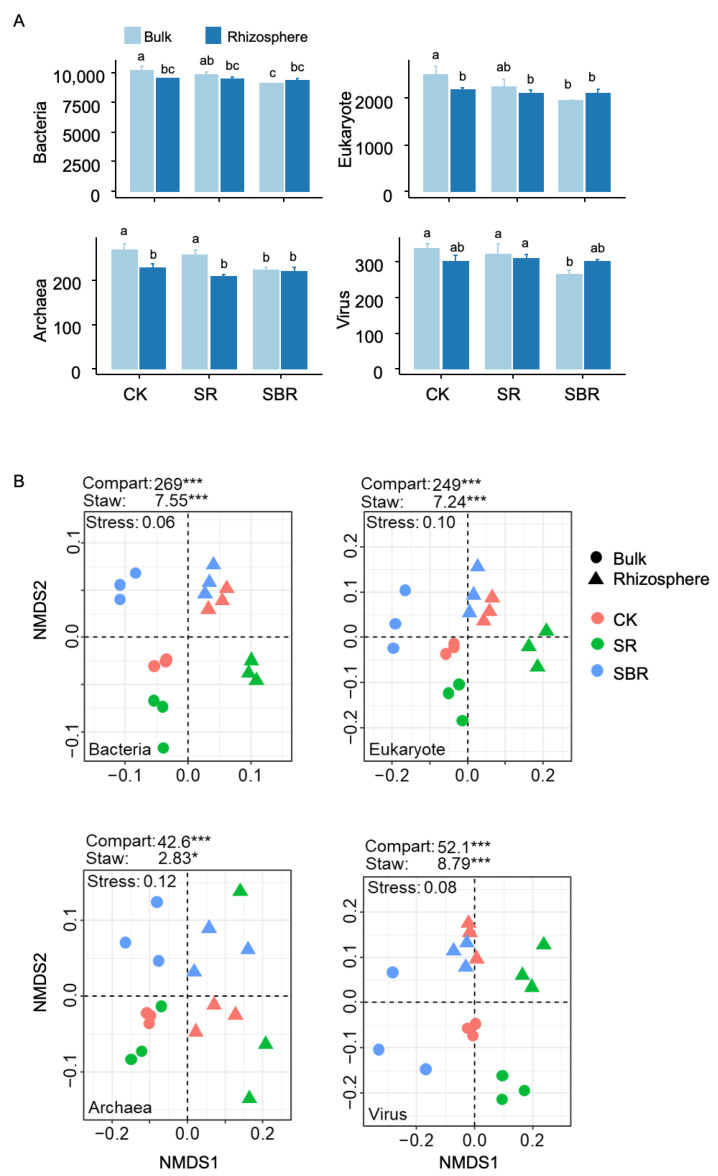
Soil diversity and community composition in rhizosphere and bulk soil. In panel (**A**), is the richness. Different letters indicate significant differences (*p* < 0.05) tested by ANOVA. In panel (**B**), the biplot of NMDS for community compositions, the contributions of compartments (bulk vs. rhizosphere) and straw application (CK, SR, and SBR) were tested by permANOVA, *, *p* < 0.05; ***, *p* < 0.001.

Compared with the CK treatment, straw returning decreased the yields of wheat by 6%, with the average values changing from 6930 to 6545 kg/hm^2^, while the straw biochar returning increased the yields to 7405 kg/hm^2^, with the increment near 7% (Table 2). The difference in taxonomic richness between bulk and rhizosphere soil was significant in the CK treatment, while the difference was not significant for SR and SBR treatments (Figure 1A). In addition, the community composition was mainly driven by the bulk and rhizosphere compartment, followed by the straw application methods (Figure 1B). 

**Figure 2:**
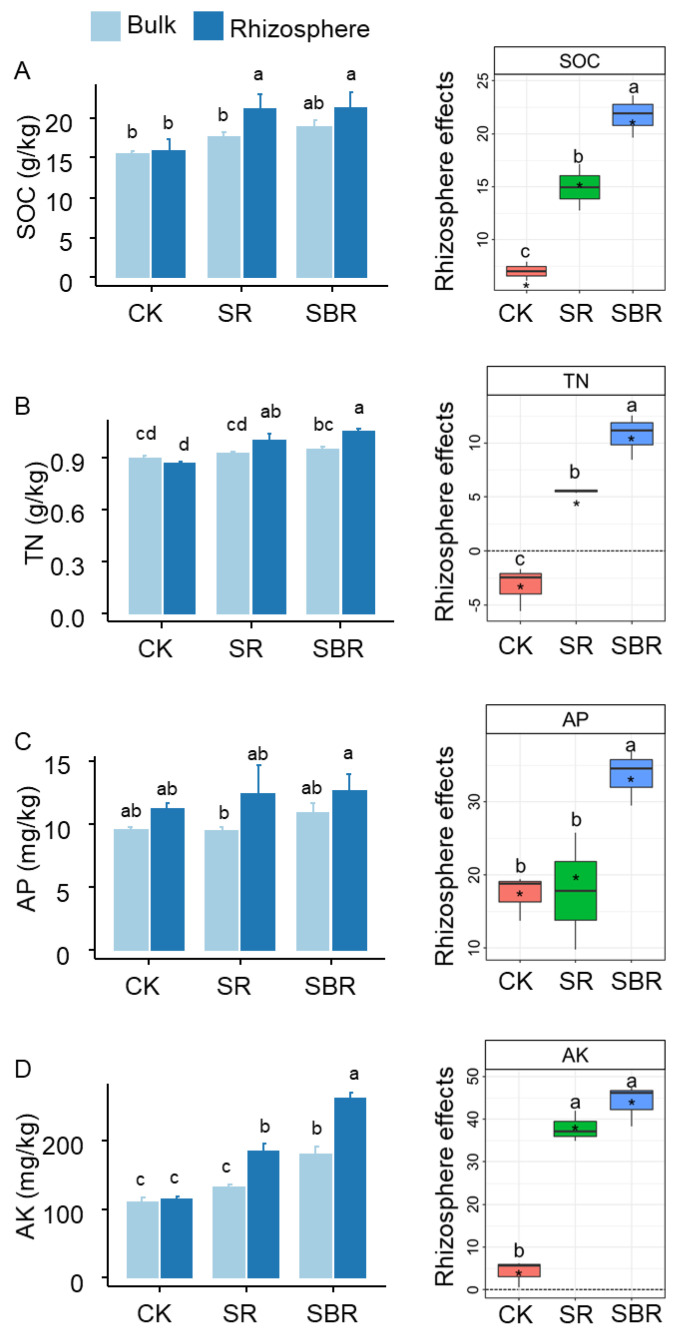
Resource availability in rhizosphere and bulk soil. (**A**) SOC, soil organic carbon; (**B**) TN, soil total nitrogen; (**C**) AP, soil available phosphorus; (**D**) AK, soil available potassium; Different letters indicate significant differences, *p* < 0.05 tested by ANOVA. The average rhizosphere effects annotated with * were significantly (*p* < 0.05) different from zero tested by the *t*-test.

Straw biochar led to the greatest rhizosphere effects on soil organic carbon (SOC, Figure 2A), soil total nitrogen (TN, Figure 2B), soil available phosphorus (AP, Figure 2C), and soil available potassium (AK, Figure 2D). In terms of the content of soil resources including carbon, nitrogen, phosphorus, and potassium, straw biochar resulted in the greatest values (Figure 2A–D).

**Figure 3:**
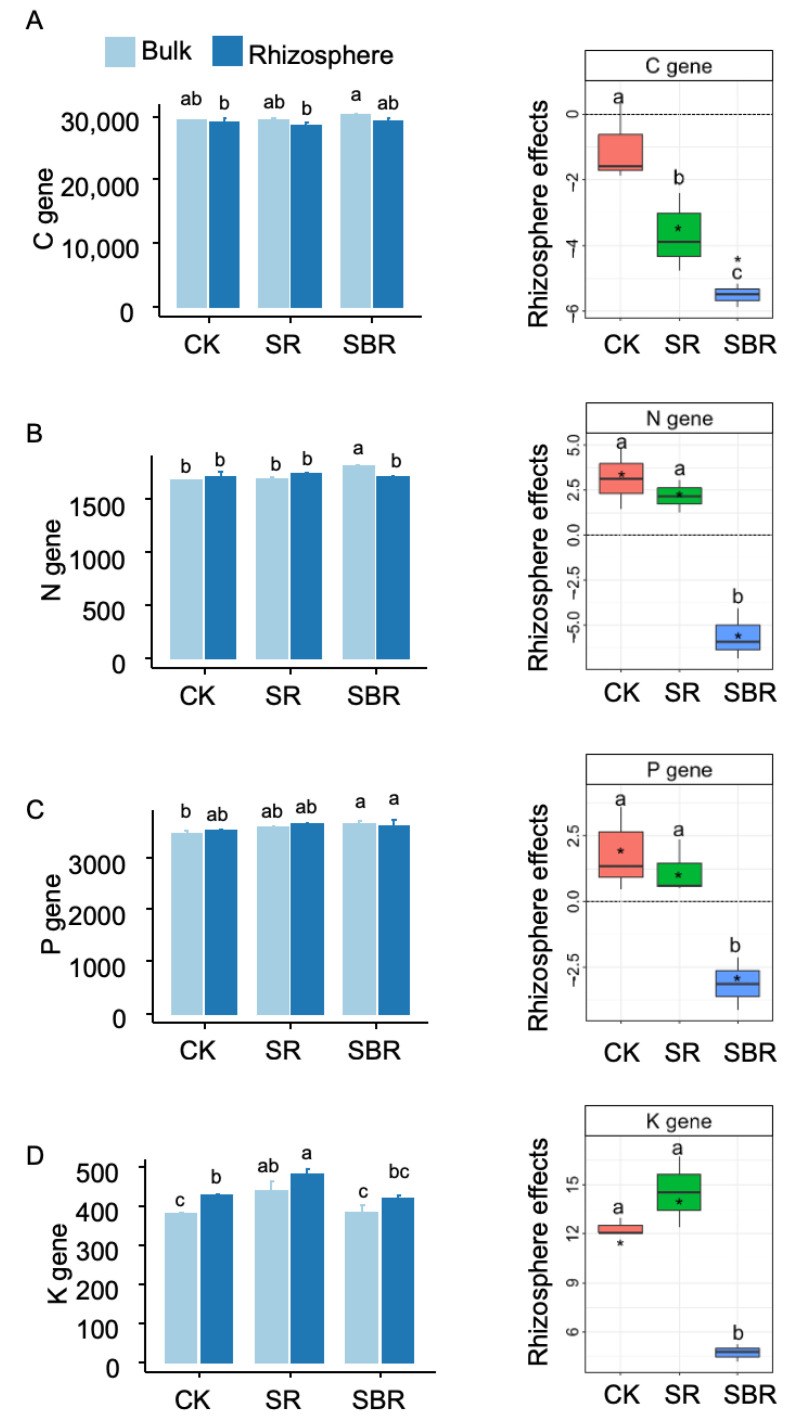
Metabolism genes in rhizosphere and bulk soil. (**A**) C gene, soil carbon metabolism genes; (**B**) N gene, soil nitrogen metabolism genes; (**C**) P gene, soil phosphorus metabolism genes; (**D**) K gene, soil potassium metabolism genes. Different letters indicate significant differences (*p* < 0.05) tested by ANOVA. The average rhizosphere effects annotated with * were significantly (*p* < 0.05) different from zero tested by the *t*-test.

Straw biochar resulted in the lowest rhizosphere effects on soil carbon genes (Figure 3A), soil nitrogen genes (Figure 3B), phosphorus genes (Figure 3C), and potassium genes (Figure 3D). With the negative values of rhizosphere effects for carbon, nitrogen, and phosphorus genes, the rhizosphere effects were positive for potassium genes (Figure 3A–D).

**Figure 4:**
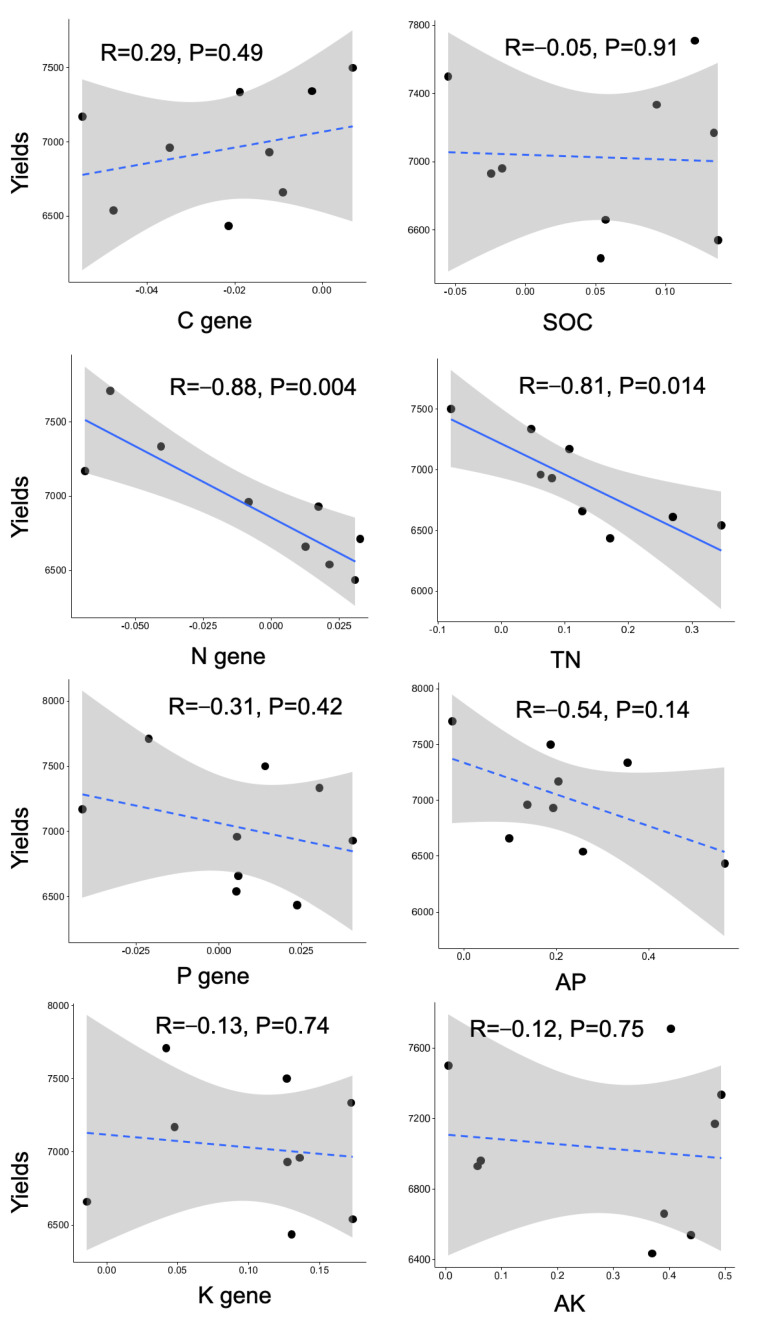
Relationships between rhizosphere effect (RE) and wheat yields. Linear regressed R and *p* values were denoted. The solid and dashed lines represent significant and nonsignificant relationships, respectively.

The relationships between crop yields and rhizosphere effects, represented by the nutrient contents and metabolism genes involved in nitrogen cycling, were mainly significant (Figure 4). Negative relationships were found for both nitrogen contents and nitrogen genes, with the R values both greater than 0.8. 

## 4. Discussion

Our results that straw biochar-driven amplification of rhizosphere effects on the diversity and community composition of the microbiome supported our hypothesis. Meanwhile, our results showed that straw biochar enhanced the rhizosphere effects on taxonomic and functional diversities in control and straw-returning treatments, with the net effects transferred from negative to positive. These results together explained the positive effects of biochar on the availability of soil resources, such as TN and AK, which showed the same trend in the present study. Biochar can increase soil microbial diversity and metabolic activities [12,28], thus resulting in positive effects on the diversity of both microbial taxa and functional genes. In addition, increases in the soil available resources might diversify the bacteria or archaea communities hosting and diversifying the virus communities [29,30]. These results highlight the comprehensive effects of biochar on the diversity of all components in soil biota. Simultaneously, the rhizosphere effects contributed to the major variation in community composition for the total gene, archaea, bacteria, and virus.

Our results showed that soil resources and metabolism genes exhibited different responses to straw biochar application. In accordance, the positive rhizosphere effects occurred for the soil resources, but the negative took place for the metabolism genes. Typically, biochar directly functions as a nutrient source and indirectly alters the contents of soil nutrients for plant roots [31]. In the present study, the straw biochar increased the contents of AP, AK, and SOC by 20~50% in the rhizosphere, which presumably benefits plant growth [32]. However, biochar application resulted in negative rhizosphere effects on nitrogen gene abundance. This might be attributed to the significant increase in biochar-driven nitrogen genes in the bulk soils compared to the rhizosphere. Higher supplies of nutrients in biochar-amended soils might suppress the abundance of microbial metabolism genes [12].

Our results that straw return resulted in a decrease in wheat yields, while the straw biochar return increased the wheat yields, indicate that there is a great advantage for the straw biochar application in agricultural ecosystems in the form of waste straw recycling [33]. We also noted that the effects of straw biochar were mainly exerted on soil resources and not on the metabolism genes. This suggests that the straw biochar might serve as a resource pool providing resources for crops in the field. It is documented that biochar is valuable as a fertilizer when resource deficiency is a major constraint on crop productivity [34]. The pyrolysis step of biochar production produces more available resources, such as potassium, in the present study. We found that the rhizosphere effects of potassium were positive while other resources were negative for biochar returning treatment. Plant roots prefer to live in biochar-amended soils, as the rhizosphere contains more biochar particles than the bulk soil [31]. Thus, the greater content of available nutrients, i.e., potassium in the present study, would be taken up by the plants and, therefore, enhance plant performance including crop yields.

## 5. Conclusions

In conclusion, using PCR-bias-free metagenomics sequencing, we found that straw biochar amendments enhanced the rhizosphere effects from wheat on soil available resources, although it suppressed the abundance of microbial metabolism genes. These microbiome variations suggest that biochar functions as a direct nutrient source rather than an indirect method of biological soil engineering in a wheat-growing agroecosystem. Thus, our study provides new insights for understanding the mechanisms of biochar as an alternative to agricultural waste recycling and a method to promote environmental safety.

## Figures and Tables

**Table 1:** Rates of NPK fertilization for each plot.

Growth Period	Crop	N—Urea (46%)	P—Diammonium Phosphate (18–46%)	K—KCl (60%)
		kg ha^−1^	kg ha^−1^	kg ha^−1^
Sowing	Wheat	183.3	130.0	100.0
Sowing	Maize	253.3	96.7	150.0
Jointing	Wheat	156.7		
Tasseling	Maize	196.7		

**Table 2:** The effect of straw application methods on the yield of wheat.

Treatment	CK	SR	SBR
Wheat yields (kg/hm^2^)	6930 ± 151 b	6545 ± 53 c	7405 ± 130 a

Note: Each value in is the mean ± SD of three replicates. Numbers followed by different lowercase letters in the same column are significantly different at *p* < 0.05. The wheat yield was calculated as kilograms per hectare (kg/hm^2^).

## Data Availability

The datasets used or analyzed during the current study are available from the corresponding author on reasonable request.

## Supplementary Materials

The following supporting information can be downloaded at: https://www.mdpi.com/article/10.3390/life13091843/s1, Table S1: Raw and clean sequence number of each sample.
