# Supplementary material for: Biochar Enhances Soil Resource Availability and Suppresses Microbial Metabolism Genes in the Rhizosphere of Wheat
Source: Life (Basel). 2023 Aug 31;13(9):1843. doi: 10.3390/life13091843 (PMC10533193; doi:10.3390/life13091843)
Supplement: Supplementary file 1 [file life-13-01843-s001.zip › life-2523347-supplementary.pdf]

Supplementary Information

Table S1 Raw and clean sequence number of each sample

Sample	Description	Number of raw sequences	Number of clean sequences	Efficiency
CK1	Replicate 1 of CK bulk soil	104,785,380	103,645,164	95.83%
CK2	Replicate 2 of CK bulk soil	115,149,386	113,839,614	95.76%
CK3	Replicate 3 of CK bulk soil	74,130,310	73,218,540	95.73%
SR1	Replicate 1 of SR bulk soil	92,040,540	90,981,848	95.73%
SR2	Replicate 2 of SR bulk soil	64,772,914	63,877,934	95.44%
SR3	Replicate 3 of SR bulk soil	65,686,088	64,878,896	95.66%
SBR1	Replicate 1 of SBR bulk soil	58,573,510	57,995,284	96.22%
SBR2	Replicate 2 of SBR bulk soil	63,519,118	62,941,894	96.34%
SBR3	Replicate 3 of SBR bulk soil	58,771,820	58,187,914	96.21%
R_CK1	Replicate 1 of CK rhizosphere soil	60,006,668	59,423,518	96.19%
R_CK2	Replicate 2 of CK rhizosphere soil	61,414,314	60,782,484	96.12%
R_CK3	Replicate 3 of CK rhizosphere soil	63,584,508	62,963,742	96.26%
R_SR1	Replicate 1 of SR rhizosphere soil	62,395,366	61,813,188	96.29%
R_SR2	Replicate 1 of SR rhizosphere soil	49,523,640	48,986,600	96.10%
R_SR3	Replicate 1 of SR rhizosphere soil	60,804,224	60,140,326	96.12%
R_SBR1	Replicate 1 of SBR rhizosphere soil	66,762,252	66,145,978	96.36%
R_SBR2	Replicate 2 of SBR rhizosphere soil	64,007,036	63,432,696	96.36%
R_SBR3	Replicate 3 of SBR rhizosphere soil	54,474,298	53,757,618	95.65%
